# Disclination mediated dynamic recrystallization in metals at low temperature

**DOI:** 10.1038/srep14215

**Published:** 2015-09-16

**Authors:** Mohammad Aramfard, Chuang Deng

**Affiliations:** 1 Department of Mechanical Engineering, University of Manitoba, 15 Gillson Street, Winnipeg, MB, R3T 5V6, Canada

## Abstract

Recrystallization is one of the most important physical phenomena in condensed matter that has been utilized for materials processing for thousands of years in human history. It is generally believed that recrystallization is thermally activated and a minimum temperature must be achieved for the necessary atomic mechanisms to occur. Here, using atomistic simulations, we report a new mechanism of dynamic recrystallization that can operate at temperature as low as T = 10 K in metals during deformation. In contrast to previously proposed dislocation-based models, this mechanism relies on the generation of disclination quadrupoles, which are special defects that form during deformation when the grain boundary migration is restricted by structural defects such as triple junctions, cracks or obstacles. This mechanism offers an alternative explanation for the grain refinement in metals during severe plastic deformation at cryogenic temperature and may suggest a new method to tailor the microstructure in general crystalline materials.

Recrystallization, which refers to the thermo-mechanical phenomenon of the formation and evolution of a network of new grains^1^,^2^, happens in crystalline materials such as minerals^3^,^4^,^5^ in geological activities and metals^2^,^6^,^7^,^8^ during deformation and heat treatment. In particular, recrystallization has been used to improve the mechanical properties of metals for thousands of years in human history e.g. through forging and annealing. As similar to other types of structural transformations in materials, conventional recrystallization is driven by thermally activated processes such as solid state diffusion, which cannot happen at low homologous temperatures. Therefore, a high temperature namely recrystallization temperature is usually required to activate recrystallization in metals, which can be achieved during hot working or annealing after cold deformation. The former is termed dynamic recrystallization (DRX)^1^,^9^,^10^ while the latter is termed static recrystallization (SRX)^1^,^6^,^8^. Owing to the high temperature, grain growth normally follows recrystallization in metals once the originally deformed grains have been all consumed by the newly formed grains.

In recent years, however, in order to obtain finer grains in materials as motivated by the “smaller is stronger” motif^11^, low temperature is being desired to prohibit grain growth^12^ during the materials processing. For example, cryogenic temperature has been applied during severe plastic deformation (SPD)^13^,^14^,^15^ to obtain ultrafine or nanostructures in bulk metals. Since the requirement for high temperature is not met, dislocation generation and evolution^16^,^17^ rather than recrystallization has been generally attributed to the grain refinement at low temperature, e.g. during SPD at cryogenic temperature. Specifically, it has been proposed that dislocation blocks (blocks or cells bounded by dislocation walls) form first at small strains during SPD. With increasing strain, the misorientation angle between neighboring dislocation blocks increases due to rotation and eventually the dislocation blocks become sub-grains^1^,^15^,^18^,^19^. On the other hand, disclination has also been proposed to cause grain refinement in metals during deformation when high temperature is not a necessity^20^,^21^,^22^,^23^,^24^. As opposed to dislocation, disclination is a type of line defect in crystals^24^,^25^,^26^ in which rotational symmetry is violated, which can be envisioned by adding (or removing) a wedge of materials to (or from) the crystal. It has been reported that a small disclination quadrupole can form at the boundary of a deforming grain and act as a nucleus of a misorientation band that can subdivide the grain^27^. Nevertheless, to date no observations of recrystallization, e.g. the nucleation of new grains directly through bulging^28^, sub-grain rotation^29^,^30^ and twinning^30^, from either experiments or atomistic simulations have been reported to occur during the deformation of crystalline materials at low temperature.

In this research, we present a series of atomistic simulations of deformation in both bicrystal and nanocrystalline Cu at various temperatures. We show that new grains can form during the deformation in those model systems by a new mechanism of DRX in terms of disclination nucleation and evolution caused by stress-driven grain boundary migration (SDGBM), which is able to operate at temperature as low as T = 10 K. Disclinations are prevalent in polycrystalline materials as the termination of grain boundaries (GBs) such as triple junctions (TJs) and are strongly correlated with GB migration^24^,^31^. For example, disclinations have been found to be responsible for the SDGBM-mediated crack healing in nanocrystalline metals^32^. This new mechanism is found to be general which relies only on common structural defects such as GBs, TJs, cracks or foreign obstacles and can occur during deformation under various loading modes.

## Results

### Disclination-mediated recrystallization in bicrystal Cu

**Figure 1:**
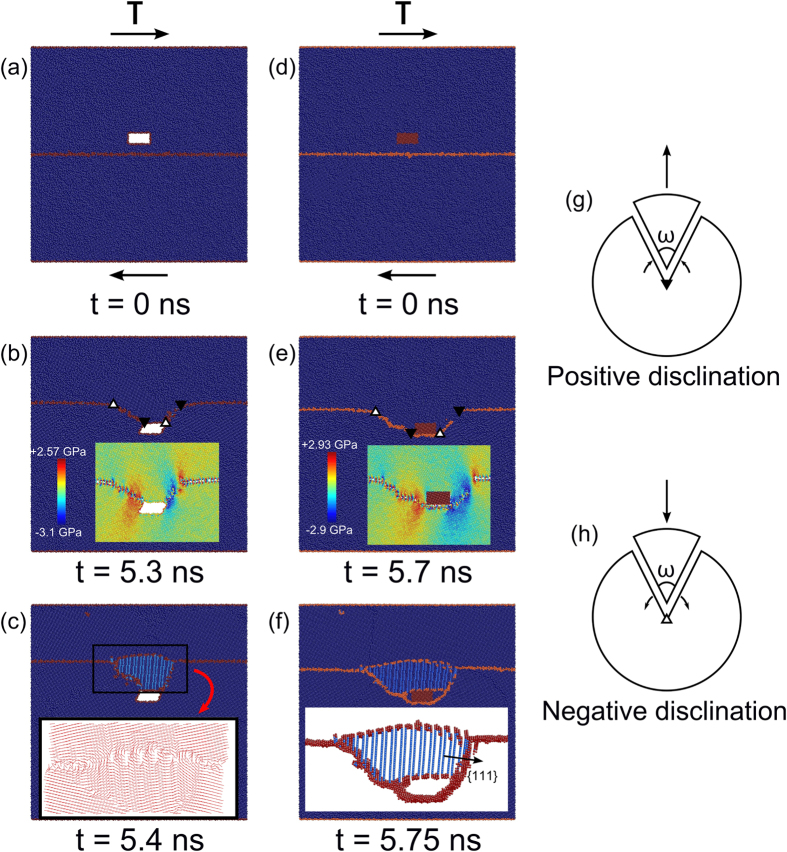
The process of LTDRX in bicrystal models with (a–c) a crack and (b–d) a rigid obstacle. (**a**,**d**) are the initial configurations. (**b**,**e**) show the formation of an asymmetric disclination quadrupole and the corresponding distribution of stress σ_zz_. (**c**,**f**) show the nucleation of the new grains. The inset in (**c**) indicates the rotation of atoms prior to the sub-grain formation and the inset of (**f**) shows the twinning in the new sub-grain. (**g**) Positive disclination with strength of ω formed by removing a wedge of material causes tensile stress, (**h**) negative disclination with strength of ω formed by adding a wedge of material causes compressive stress.

The key process in the disclination-based low temperature DRX (LTDRX) mechanism is SDGBM in presence of restrictions such as TJs, cracks or foreign obstacles, which are common defects in crystalline metals. As similar to previous studies on SDGBM^33^, a simple bicrystal model of Cu with periodic boundary conditions applied to the directions parallel to the GB plane was constructed so that the results would be general and not be specific to a certain grain size. As a model system, the bicrystal model shown in Fig. 1 contains a special Σ17 (350) symmetric tilt GB with a misorientation angle of 61.93^o^. A crack (Fig. 1(a)) or a precipitate (Fig. 1(d)) was created in the simple bicrystal model, which was then deformed by simple shear. Due to shear coupling effect that has been revealed in previous works^33^,^34^,^35^, the GB moved upward towards the crack or the precipitate. As shown in Fig. 1(b), when the GB reached the crack, it partially passed the crack with the central part bulged and pinned at the crack ends after 5.3 *ns* of the deformation. Furthermore, the inset of Fig. 1(b) shows the distribution of stress σ_zz_ around the crack and the bulged GB, which clearly indicates the formation of an asymmetric disclination quadrupole^32^. As the deformation continued, the strength and associated strain energy of this disclination quadrupole increased until a threshold was reached, at which the atoms within it rotated dramatically and a new sub-grain was nucleated (Fig. 1(c)). As indicated in the inset of Fig. 1(c), the atoms confined by the disclination quadrupole experienced significant clockwise rotation before the new grain was formed. Similar process of a new grain formation was observed in the bicrystal model containing a rigid precipitate. The precipitate was created as a group of atoms that moved as a rigid body (Fig. 1(d)). It can be seen in Fig. 1(e) that as similar to the crack, the precipitate also bent and pinned the moving GB, causing the formation of a disclination quadrupole which is evident from the distribution of stress σ_zz_ and marked by the symbols of open and solid triangles. When the energy of this quadrupole reached a certain value, a new sub-grain with significant twinning was formed, as shown in Fig. 1(f). Since the new grain nucleation process shown in both bicrystal models occurred at 10 K, the mechanism is considered to be LTDRX.

### Disclination-mediated LTDRX in Cu during general SDGBM

**Figure 2:**
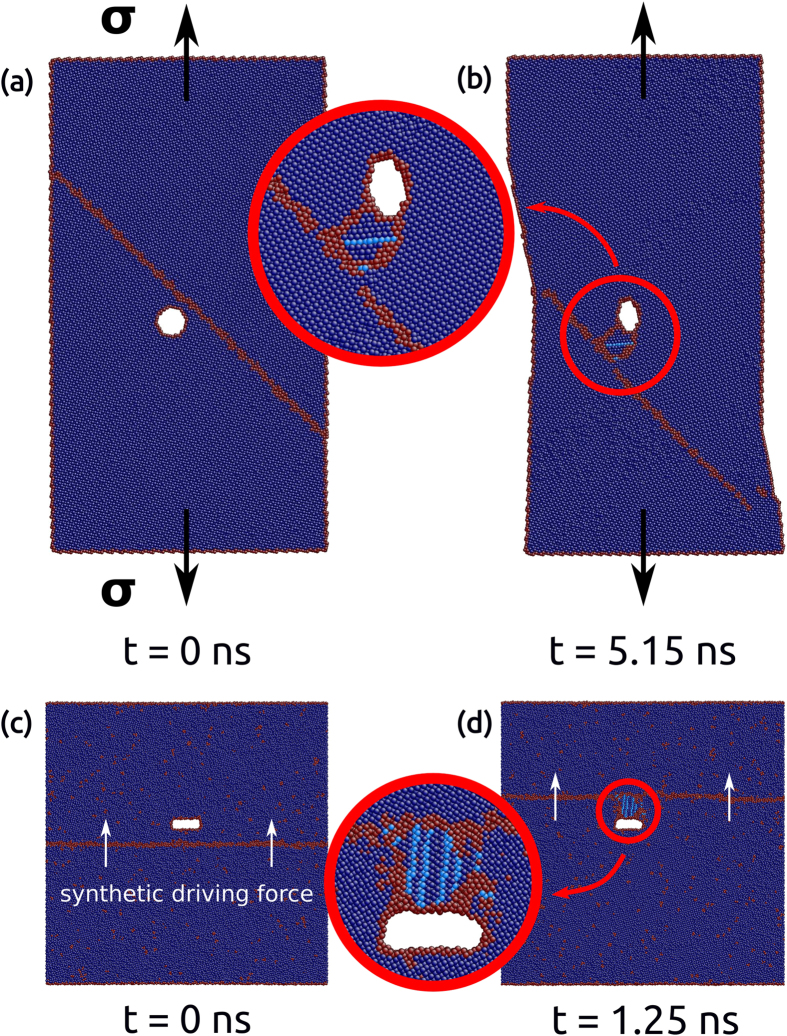
(**a**) Undeformed and (**b**) deformed bicrystal model under tension at 10 K. (**c**) Undeformed and (**d**) deformed bicrystal model with imposed synthetic driving force of 0.015 *e*V*/atom* at 600 K.

The disclination-mediated LTDRX was further investigated by applying two additional loading modes, *i.e.* tension and synthetic driving force^36^ to the bicrystal model containing a crack, as shown in Fig. 2. Specifically Fig. 2(a) shows the bicrystal model consisting of a Σ17 (350) GB that is inclined at 45^o^ under tension at 10 K. The GB is inclined so that the applied tension would impose a shear stress on the GB. It can be seen in Fig. 2(b) that the crack caused the moving GB to bend which resulted in the formation of a new grain. As similar to the observations in Fig. 1, coherent twin boundaries were observed in the newly formed sub-grain. On the other hand, Fig. 2(c) shows the model to study the SDGBM due to an applied synthetic driving force of 0.015 *e*V*/atom* at 600 K. A new grain with coherent twin boundaries was also formed when the GB passed the crack as shown in Fig. 2(d). The results in Fig. 2(d) suggest that the disclination-mediated DRX also operates at relatively high temperatures.

### Atomistic mechanisms of LTDRX in Cu

**Figure 3:**
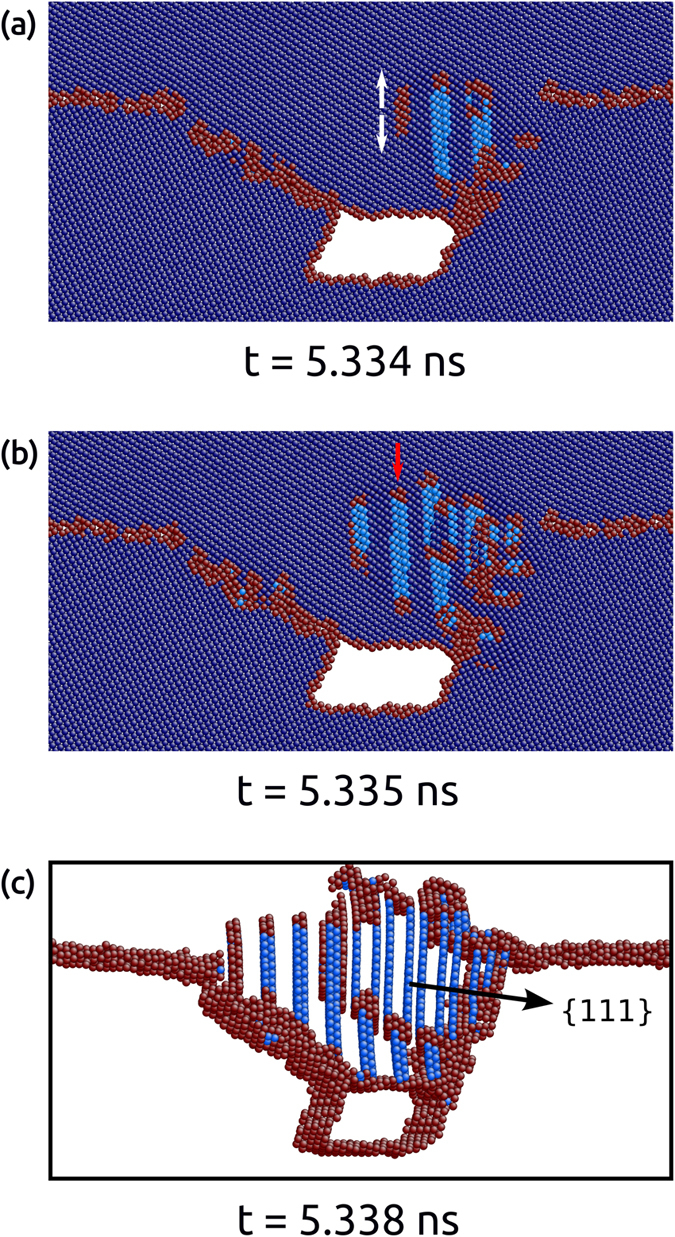
Detailed atomistic mechanisms of atom rotation and new grain nucleation in the bicrystal model with a crack under shear deformation. (**a**) Dislocation nucleation at the onset of atom rotation, (**b**) propagation of the nucleated partial dislocations, and (**c**) twinning upon further rotation.

To clarify the detailed atomistic mechanism of bulging, sub-grain rotation and twinning, a series of snapshots at intermediate steps between Fig. 1(b,c) are shown in Fig. 3. As shown in Fig. 3(a), at the onset of the sub-grain nucleation, Shockley partial dislocations nucleated in the area adjacent to the disclination quadrupole. It is important to emphasize that the dislocation nucleation did not start from the crack surface or GBs, which are the normal dislocation nucleation sites in crystalline metals during plastic deformation. On the contrary, the dislocations were nucleated from within the region surrounded by the disclination quadrupole, where the most significant rotation would be expected. Therefore, in contrast to previous models of SPD^22^ or DRX^15^,^37^,^38^ in which dislocation activities preceded the new sub-grain formation, the dislocations observed in Fig. 3 were the product rather than the cause of the atom rotation and the subsequent new grain formation. Although sometimes the dislocation-based model was also referred as LTDRX^39^, it is important to note that the characteristics of DRX consisting of bulging, sub-grain rotation and twinning was missing in these studies.

Furthermore, Figure 3(b) shows that the nucleated dislocations propagated in both upward and downward directions. As the rotation of atoms continued, another set of Shockley partial dislocations nucleated and propagated on {111} planes adjacent to the pre-formed stacking faults and transformed them to {111} coherent twin boundaries (Fig. 3(c)). The same mechanism of new grain and twin boundary formation has also been observed at higher temperature, *i.e.* 400 K, in both bicrystal models with a crack or rigid precipitate. This observation is consistent with previous analytical models on disclination-mediated twinning in metals^27^,^40^ that the formation of coherent twin boundaries following partial dislocation nucleation can significantly reduce the strain energy of the material^41^, thus accommodating the nucleation of new sub-grains.

### Energy and Stress Analysis

While the bulging and serration of the GB during LTDRX found in Fig. 3 is consistent with previous findings regarding the formation of “serrated” GBs due to SDGBM, e.g. in Al-5% Mg^18^, it is important to note that the new mechanism of LTDRX observed in this work is disclination rather than dislocation based. Furthermore, in contrast to the DRX found by Miura *et al.*^42^ in bicrystal Cu under tension that the newly formed sub-grains were all special twin grains, the LTDRX mechanism proposed in this work tends to nucleate general grains.

**Figure 4:**
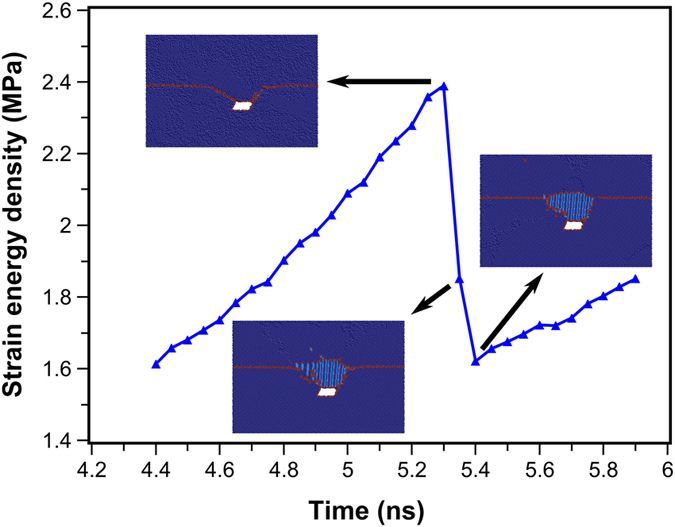
Evolution of the elastic strain energy density of the bicrystal model containing a crack under shear deformation at 10 K.

In order to further justify the atomistic mechanisms proposed above, the energy favorability of the disclination-mediated LTDRX was considered. Here the elastic strain energy of atoms before and after the nucleation of the sub-grain was calculated by using equation (1) and the constants for copper proposed in^43^:


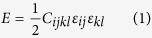


where *C*_*ijkl*_ are elastic constants and *ε*_*ij*_ and *ε*_*kl*_ are strains with i, j, k, l = 1–3. As a representative example, the time evolution of the elastic strain energy density of the bicrystal model containing a crack during the SDGBM is plotted in Fig. 4. It was found that the energy of the system first increased monotonically with time (or the applied shear strain) until the new grain was nucleated, after which the system energy experienced a sudden drop up to 0.8 MPa. This drop of energy is comparable to the energy per unit area of usual GBs which is in the order of ~1 MPa^44^. This observation is in excellent agreement with Poliak and Jonas^45^ that DRX can be initiated at any temperature when the stored local energy reaches a maximum and the dissipation rate reaches a minimum.

**Figure 5:**
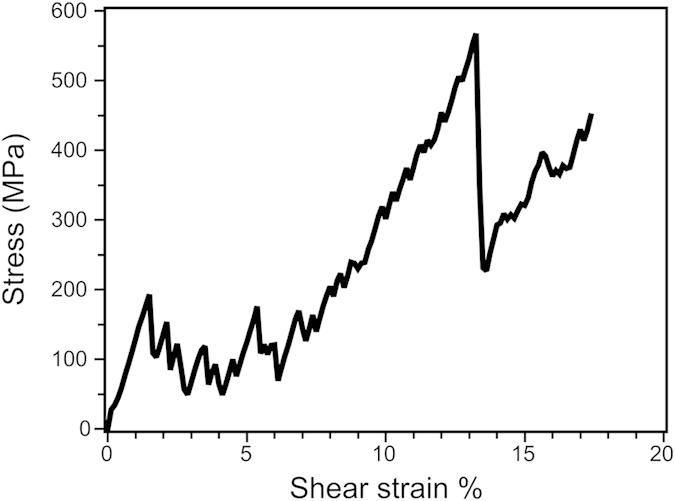
Shear stress vs. shear strain in the bicrystal model with a crack at 10 K.

Furthermore, the corresponding shear stress-shear strain curve shown in Fig. 5 can be well understood by the different stages of SDGBM. As having been reported previously, the shear stress first monotonically increased with the shear strain until the stress became so high that the GB started to move, e.g., at 1.4% strain^33^. Once the GB started moving, a stick-slip behavior occurred, which is also consistent with previous studies^33^. When the GB reached the crack and was pinned, the SDGBM was hindered and the GB became bulged. This section can be seen from the monotonic increase in stress from ~7% strain until the new grain was formed at 13.3% strain. It is also worth noting that the stress increase during GB bulging was mostly accumulated near the crack region which facilitated the sub-grain formation.

### Disclination-mediated recrystallization in nanocrystalline Cu

**Figure 6:**
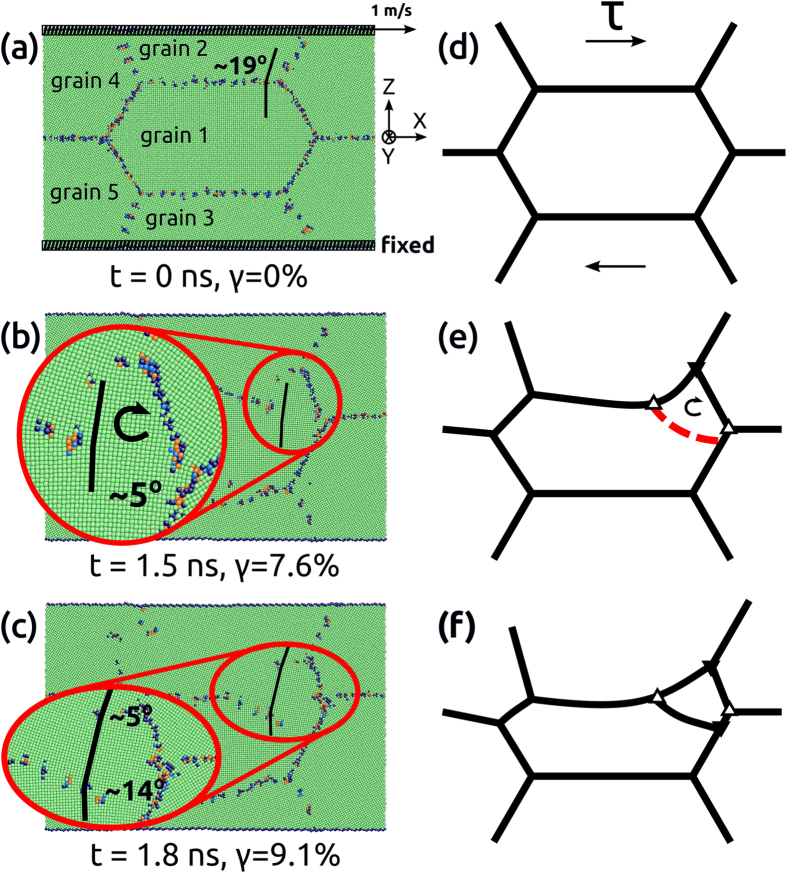
The LTDRX process in a honeycomb nanocrystalline model of Cu under shear deformation at T = 200 K. (**a**) The initial configuration, (**b**) the configuration showing the nucleation of the new sub-grain at 1.5 *ns* or 7.6% shear strain, (**c**) the configuration showing the rotation of the newly formed sub-grain at 1.8 *ns* or 9.1% shear strain. (**d,e,f**) Schematic model describing the LTDRX process mediated by disclinations. The insets in (**b,c**) show the zoomed view of the new sub-grain, and the solid and open triangles in (**e,f**) show the disclination characteristics of the TJs and the bulged GB.

As similar to cracks or precipitates, TJs can also serve as pinning obstacles to SDGBM in metals^35^,^46^. In particular, as the grain size was reduced to nanometer scale, the volume fraction of TJ increased dramatically and the pinning effects of TJs may become significant. In order to investigate if TJs alone can also lead to the disclination-mediated LTDRX mechanism, a quasi-3-dimensional nanocrystalline model made of copper with honeycomb grains and periodic boundary conditions in x- and y-directions (Fig. 6(a)) was built and deformed by shear at T = 200 K. In particular, the y-direction is parallel to <0 0 1> direction so that the model can represent a textured system with columnar grains. It can be seen in Fig. 6(b) that the GB between grains 1 and 2 moved downward while being constrained by two TJs, resulting in a bulged geometry of the moving GB. This observation was consistent with previous findings^35^ that the TJs can retard the SDGBM and bend the GB. It was further found that as the GB continued migrating, the atoms confined by bulged GB and nearby TJ rotated clockwise until a new sub-grain was formed. Figure 6(c) shows that the newly formed sub-grain rotated during the subsequent loading as indicated by the change of misorientation angles between the new and neighboring grains. A similar process was also found when the temperature changed to T = 400 K.

It is important to note that no dislocations were involved during the LTDRX process; the first dislocations appeared at *t* = 2.4 *ns* when the new grain was already formed. More details of the sub-grain nucleation and rotation process can be found in Movie S1 of the Supplementary Material. This process can also be described by the disclination-mediated LTDRX model; a schematic of this mechanism is shown in Fig. 6(d–f). In Fig. 6(d), a grain surrounded by six GBs and TJs is under shear deformation. Assuming that the top GB moves down due to SDGBM as similar to Fig. 6(b), the moving GB will bulge as shown in Fig. 6(e) due to the asymmetric pinning effects imposed by the two TJs that bound the GB^35^. Specifically, the TJ on the left side will move along with the GB whereas the TJ on the right side will stay behind. The origin of such asymmetric pinning effects of the two TJs during SDGBM has been studied previously^35^. Consequently, the strength of the disclination on the right side increases, as indicated by the top solid triangle in Fig. 6(e). Meanwhile the bulging of the moving GB causes a partial disclination to form at the rotating point, as indicated by the top open triangle in Fig. 6(e). As the migration and bulging of the GB continues the strength and strain energy of the disclinations increases, which causes the atoms confined by the bulged GB and TJ to rotate. When the strain energy reaches a threshold, the atoms between the two disclinations rotate significantly and form a new sub-grain. The new sub-grain adds another disclination to the system and forms an asymmetric disclination quadrupole, which imposes further rotation of the newly formed sub-grain, Fig. 6(f).

## Discussion

Since TJs, cracks and precipitates are all common structural defects, it is expected that disclinations and LTDRX play important roles in the overall microstructural evolution in crystalline metals under deformation at low to medium temperatures. For example, the LTDRX mechanism observed in the nanocrystalline model can explain grain refinements in newly reported cryo-indentation of nanocrystalline Cu, in which it has been reported that untwined nanocrystalline Cu exhibited grain distortion and refinement under indentation at 77 K^47^. Nevertheless, it is important to note that the overall microstructural evolution in metals during severe deformation is very complicated which involves multiple mechanisms. For example in contrast to the cryo-indentation of nanocrystalline Cu which showed grain refinement^47^, Zhang *et al.* observed rapid grain growth in ultra-fine and nanocrystalline Cu under indentation^38^. While dislocation activities are almost inevitable under experimental conditions, it is the purpose of this work to propose that the disclination-mediated LTDRX mechanism may have been overlooked and could be used to tailor material structures under some special circumstances, e.g., when cracks or precipitates are abundant.

However, it should be cautious that the disclination-based LTDRX mechanism strongly depends on the types of GBs and the configuration of the grain structures. By simulating bicrystal Cu systems containing different types of GB it was found that SDGBM did not always lead to the formation of disclination quadrupoles and new sub-grains. Part of the reason is that SDGBM strongly depends on the tilt axis and the misorientation angle and is only significant for certain types of symmetric GBs^33^. On the other hand, while it may seem uncommon to have no dislocation nucleation during the simulation of such large deformation in nanocrystalline Cu, the phenomenon may be connected with the special configuration of the simulated nanocrystalline model (Fig. 6). For comparison, we have simulated shear deformation in nanocrystalline Cu constructed by Voronoi tessellation with dimensions and average grain size similar to that in Fig. 6 but with random grain shape and orientation. In these simulations, dramatic dislocation activities and grain rotation were observed.

Furthermore, although SDGBM has been rarely connected with disclinations in previous studies, some disclination-based models have already been proposed to describe the plastic deformation and grain refinement in metals during deformation^20^,^21^,^23^,^24^,^48^,^49^,^50^. For example, Orlova *et al.* have proposed that an asymmetric disclination quadrupole can cause the region inside it to rotate, leading to the formation of two diagonal boundaries and dividing the grain into four new sub-grains^20^. In this work, however, it was found that a disclination quadrupole does not necessarily nucleate dislocations in a cross shape as proposed in^20^ but causes a uniform lattice rotation.

In summary, a new mechanism of LTDRX was found based on molecular dynamics simulations in crystalline Cu during deformation. The mechanism relied on the generation of disclination quadrupoles by SDGBM with restrictions by TJs, cracks or foreign obstacles. The disclination quadrupoles can induce dramatic rotation of the atoms within them and ultimately the nucleation of new sub-grains. This mechanism was found to be general, i.e. insensitive to temperature and independent of the loading mode, which may contribute to the grain refinement in metals during SPD at cryogenic temperatures. The novel mechanism of disclination-induced LTDRX may be used to design new methods of tailoring the microstructure in general crystalline materials.

## Methods

LAMMPS^51^ with embedded atom method potential for Cu^52^ was used for all simulations. The dimensions of the model shown in Fig. 6(a) are 30 *nm* × 2.5 *nm* × 20 *nm* in x-, y- and z- directions, respectively. The sizes of grains 1, 4 and 5 are 20 *nm* × 2.5 *nm* × 10 *nm* and the sizes of grains 2 and 3 are 20 *nm* × 2.5 *nm* × 5 *nm*.The dimensions of bicrystal models in Fig. 1 are 40 *nm*×1.8 *nm* × 40 *nm* and the size of the crack or precipitate in Fig. 1 is 4 *nm* × 1.8 *nm* × 2 *nm*. The boundary conditions in x- and y-directions are periodic while the two surfaces perpendicular to z-direction are set to free in all models. The visualization was realized by using AtomEye^53^ with Ackland and Jones’ analysis^54^. The orientation of each grain in the nanocrystalline Cu model is tabulated in Table 1.

Before applying shear deformation, the system was relaxed at desired temperature using Nosé-Hover thermostat^55^,^56^ under isothermal-isobaric ensemble (NPT). In order to apply shear, two thin slabs of atoms (0.5 *nm* thickness each) were defined on top and bottom of the model to move as rigid body. The bottom slab was fixed and the top slab was translated at constant velocity of 1 *m/s* along x-direction, which induced strong SDGBM in the model due to shear coupling effects^33^. During the shear deformation the temperature was kept constant using canonical thermal ensemble (NVT). The stress was computed by using Virial stress equations^57^.

## Additional Information

**How to cite this article**: Aramfard, M. and Deng, C. Disclination mediated dynamic recrystallization in metals at low temperature. *Sci. Rep.* **5**, 14215; doi: 10.1038/srep14215 (2015).

## Supplementary Material

Supplementary Information

Supplementary Video

## Figures and Tables

**Table 1:** The lattice orientations of each grain in the model shown in Fig. 1(a).

Grain number	X	Y	Z
1	[100]	[010]	[001]
2	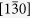	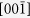	[310]
3	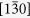	[001]	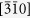
4	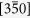	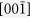	[530]
5	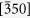	[001]	[530]
