# Supplementary material for: Disclination mediated dynamic recrystallization in metals at low temperature
Source: Sci Rep. 2015 Sep 16;5:14215. doi: 10.1038/srep14215 (PMC4570976; doi:10.1038/srep14215)
Supplement: Supplementary Information [file srep14215-s1.pdf]

Supplementary Information for

Disclination mediated dynamic recrystallization in metals at low temperature

Mohammad Aramfard and Chuang Deng*

Department of Mechanical Engineering, University of Manitoba

15 Gillson Street, Winnipeg, MB R3T 5V6, Canada

**E-mail: dengc@ad.umanitoba.ca*

Movie S1: The detailed process of new-grain formation in nanocrystalline Cu under shear deformation at 10 K.
